# Use of Wild Rice (*Zizania palustris* L.) in Paddy-Scale Bioassays for Assessing Potential Use of Mining-Influenced Water for Irrigation

**DOI:** 10.1007/s10230-022-00908-0

**Published:** 2022-11-18

**Authors:** O’Niell R. Tedrow, Peter F. Lee

**Affiliations:** 1 Northeast Technical Services, Inc., 526 Chestnut Str, Virginia, MN 55792 USA; 2 Vermilion College, 1900 East Camp Str, Ely, MN 55731 USA; 3 Centennial Building, Lakehead University, 955 Oliver Rd, Thunder Bay, ON P7B 5E1 Canada

**Keywords:** Water resources, Sulfate, Sulfide mining

## Abstract

As surface water resources become more intensely used, and occasionally non-useable, consideration of non-conventional water resources for anthropogenic use has become more prevalent. Potentially critical non-conventional water sources include flooded mine-pit lakes. However, water in these lakes can contain potentially problematic concentrations of contaminants of concern. We evaluated the potential use of elevated sulphate (SO_4_) mining-influenced waters with low to non-detect metals concentrations for irrigation of wild rice (Zizania palustris L.; WR), a culturally and economically important species. Two flow-through in-situ paddies were developed adjacent to two mine-pit lakes with differing chemical water characteristics; specifically, Pit A contained ≈350 mg SO4 L^−1^ and Pit C contained ≈1350 mg SO4 L^−1^. Throughout the course of multiple consecutive growing seasons, no adverse WR responses to these mining-influenced water exposures were observed. Based on data and observations from this study, potential use of mining-influenced waters containing elevated SO_4_ as the primary contaminant for appropriate irrigation purposes is supported. However, site-specific conditions and potential environmental risks must be considered prior to use of mining-influenced waters for anthropogenic applications.

**Supplementary Information:**

The online version contains supplementary material available at 10.1007/s10230-022-00908-0.

## Resumen

A medida que los recursos hídricos superficiales se utilizan más intensamente, y que en ocasiones no se pueden utilizar, la consideración de los recursos hídricos no convencionales para uso antropogénico se ha hecho más frecuente. Entre las fuentes de agua no convencionales potencialmente críticas se encuentran los lagos inundados de las minas. Sin embargo, el agua de estos lagos puede contener concentraciones potencialmente problemáticas de contaminantes preocupantes. Se evaluó el uso potencial de aguas impactadas por la minería con elevados niveles de sulfato (SO4) y concentraciones de metales bajas o no detectables para el riego de arroz silvestre (Zizania palustris L.; WR), una especie de importancia cultural y económica. Se desarrollaron dos arrozales de flujo in situ adyacentes a dos lagos de minas con diferentes características químicas del agua; concretamente, el lago del pit A contenía ≈350 mg L-1 y el del pit C contenía ≈1.350 mg L-1 de sulfato. A lo largo de múltiples temporadas de cultivo consecutivas, no se observaron respuestas adversas de WR a estas exposiciones de agua. Basándose en los datos y observaciones de este estudio, se apoya el uso potencial de las aguas impactadas por la minería que contengan SO4 como contaminante primario para fines de riego apropiados. Sin embargo, antes de utilizar las aguas influidas por la minería para aplicaciones antropogénicas deben considerarse las condiciones específicas del lugar y los posibles riesgos medioambientales

## Abstract (de)

Da die Oberflächenwasserressourcen immer intensiver genutzt werden und gelegentlich nicht mehr nutzbar sind, werden unkonventionelle Wasserressourcen für den menschlichen Gebrauch immer wichtiger. Zu den unkonventionellen Wasserressourcen gehören geflutete Grubenseen. Das Wasser in diesen Seen kann problematische Konzentrationen an bedenklichen Schadstoffen enthalten. Wir untersuchten die potentielle Nutzung von Grubenwasser mit erhöhter Sulfatkonzentration (SO_4_) und niedrigen bis nicht nachweisbaren Metallkonzentrationen für die Bewässerung von Wildreis (Zizania palustris L.), einer kulturell und wirtschaftlich wichtigen Art. Neben zwei Grubenseen mit unterschiedlichen hydrochemischen Wassereigenschaften wurden zwei Versuchsfelder angelegt. Grube A enthielt ≈ 350 mg/L und Grube C enthielt ≈ 1.350 mg/L Sulfat. Im Laufe mehrerer aufeinanderfolgender Vegetationsperioden wurden keine nachteiligen Reaktionen des Wildreises auf die bergbaubedingte Exposition beobachtet. Auf der Grundlage der Daten und Beobachtungen dieser Studie wird die Verwendung von bergbaubeeinflussten Wässern mit erhöhten Sulfatkonzentrationen für Bewässerungszwecke befürwortet. Vor der Verwendung müssen die standortspezifischen Bedingungen und die potentiellen Umweltrisiken betrachtet werden.

## Introduction

Mining activities throughout the world influence water quality even as water shortages become increasingly more common in many locations (Miller 2006; Northey et al. 2013; Liu et al. 2021). As such, strategies are being developed to identify beneficial mining-influenced water use and reuse opportunities (Apostu et al. 2020; Doupe’ and Lymbery 2005; Gunson et al. 2012; Jones 2012; McCullough et al. 2020; Schultze 2012; Schultze et al. 2022) including aquaculture (Axler et al. 1992, 1996; McNaughton and Lee 2004), recreational and industrial uses (McCullough and Lund 2006), irrigation of some crops (Annandale et al. 2002, 2009, 2017, 2018, 2019, 2021), and sources of potable water (APUC 2021; VPUC 2020). However, mining-influenced water can be associated with concentrations of potentially toxic elements which may be of concern in areas of use (Doupe’ and Lymbery 2005; Gerdol et al. 2018; Herbert et al. 2015; Karanthasis and Johnson 2003; Khan et al. 2005; Kumar et al. 2009; McCullough and Lund 2006; Miller et al. 1996; Nordstrom 2011).

Concentrations of potentially toxic elements such as Al, Cu, and Zn found in other locations (Nordstrom 2011) are not observed in most mining-influenced waters in Minnesota (MN, USA). Instead, sulphate (SO_4_) from mining activities entering wild rice (WR) waters is the primary concern in MN. Use of WR in aquatic bioassays has increased in recent years in an effort to better understand how exposures of SO_4_ (> 10 mg L^−1^; current MN WR water quality criterion) may influence phenology, distribution, and productivity. Laboratory, and small-scale field, investigations have focused on measuring responses of WR to well-defined exposures of SO_4_ and hydrogen sulphide (H_2_S) associated with mining-influenced waters (Fort et al. 2014, 2017; Pastor et al. 2017; LaFond-Hudson et al. 2018, 2020). Development and use of these paddy-scale bioassays was critical to better understanding larger-scale and longer-term in-situ responses of WR to exposures of elevated SO_4_ (≈350 and 1350 mg L^−1^) in mining-influenced waters under a more realistic scenario. Critically, these paddies represent, on a smaller-scale, commercial paddy WR production.

### WR Water Review

Various surveys have examined water quality in lake-grown WR. In a survey of WR lakes in MN and Ontario, Lee (1979) observed that most lakes supporting WR had an average alkalinity of ≈40 mg L^−1^, and average pH values of ≈6.9. Pip (1984) examined the distribution of 59 species of aquatic macrophytes in Manitoba, including WR, concluding that SO_4_ concentrations were of minor importance for WR distribution. Pillsbury and McGuire (2009) attributed losses of WR in MN and Wisconsin to increased ammonia, pH, water depth, and residential and agricultural developments in study areas. Jorgenson et al. (2013a) showed that WR could grow in waters with seasonal total P concentrations reaching 1500 µg L^−1^. Although WR distribution may be at least correlated to water chemistry, WR also influences chemical characteristics of water in which it lives. Lee and McNaughton (2004) showed that water surrounding WR stands contained lower SO_4_, and higher conductivity, Ca, and Fe concentrations than adjacent open-water areas.

One concern with use of some mining-influenced waters for WR irrigation is exposure to potentially toxic elements. Bioassays conducted by Lee and Hughes (1998) determined concentrations (≥1.0 mg L^−1^) of Al, Cd, Cu, Hg, and Pb in water that were detrimental to early WR development. An additional concern with using mining-influenced water for WR irrigation is a possible effect from SO_4_ (Moyle 1944, 1945, 1956). Moyle documented WR was primarily found in waters with a SO_4_ concentration of less than 10 mg L^−1^. However, WR was also observed growing in waters ranging from 2- > 200 mg SO_4_ L^−1^ (Lee and Hughes 2000; Lee and Stewart 1981; Moyle 1945; Paulishyn and Stewart 1970; Rogalski et al. 1971). In a comprehensive field study completed by the MN Pollution Control Agency (MPCA) during 2011–2013, WR was observed growing in surface waters ranging from < 0.5–838 mg SO_4_ L^−1^. More recent microcosm studies concluded aqueous SO_4_ may not be a primary chemical characteristic adversely influencing WR phenology; rather, pore water (PW) H_2_S may be more adversely influential on WR plant development (Myrbo et al. 2017; Pastor et al. 2017; LaFond-Hudson et al. 2018, 2020). Fort et al. (2017) concluded that iron (II) complexation with sulphide may decrease adverse WR responses to these sulphide exposures. In a bucket-style mesocosm study in MN, LaFond-Hudson et al. (2018, 2020) inferred FeS coatings on WR roots may have resulted in decreased plant height, dry weight biomass (DWB), decreased overall developmental rate, and seed characteristics including per-plant productivity. It was hypothesized that more realistic assessments of the importance of SO_4_ and H_2_S exposures to WR could be achieved using paddy-scale bioassays and longer-term in-situ exposures of elevated SO_4_ (≈350 and 1,350 mg L^−1^) in mining-influenced waters. Detailed descriptions of WR paddy cultivation include Oelke (2006), Oelke et al. (1982, 1997), and Marcum and Porter (2006), with foci on nutrient amount and timing requirements (Grava 1977; Grava and Raisanen 1978; Grava and Rose 1975; Oelke et al. 1982, 1997; Sims et al. 2012a, b). Nitrogen deficiency has been suggested as a particular problem for growing WR in mining-influenced sediments (Tedrow 2020; Tedrow and Lee 2021).

## Materials and Methods

### Paddy Design, and Inflow/Outflow and Substrate Sampling

In an effort to quantify influences of elevated SO_4_ from mining-influenced waters on WR growth and development, flow-through WR paddies were constructed adjacent to two mine pit lakes ≈2.5 km apart (Paddy A adjacent to Pit A; Paddy C adjacent to Pit C). Water contained within the two pits is sufficiently different to encompass a range of aqueous SO_4_ [≈350 mg L^−1^ (Pit A) to 1,350 mg L^−1^ (Pit C)] theorized to adversely influence WR distribution, phenology, and productivity. Water depth (Thomas and Stewart 1969) and competing vegetation were mitigated as adverse influence variables (Vicario and Halstead 1968; Stevenson and Lee 1987; Elakovich and Wooten 1989; Quayyum et al. 1999; Tucker et al. 2011).

Wild rice (WR; *Zizania palustris* L.) was chosen as the aquatic test species for this work due to its cultural and economic importance to the NE region of MN; and for its overall phenology. WR is an aquatic annual, and therefore must produce viable seeds each year to have re-growth the following year. Initiating/planting paddies of WR is also made simpler by this fact—broadcasting grain is less resource intensive than individual cutting, rhizome, or root-ball planting. Additionally, potential vegetation management (removal of competing vegetation; adding viable seed; substrate nutrient management) was simplified through use of this annual aquatic grain.

**Fig. 1:**
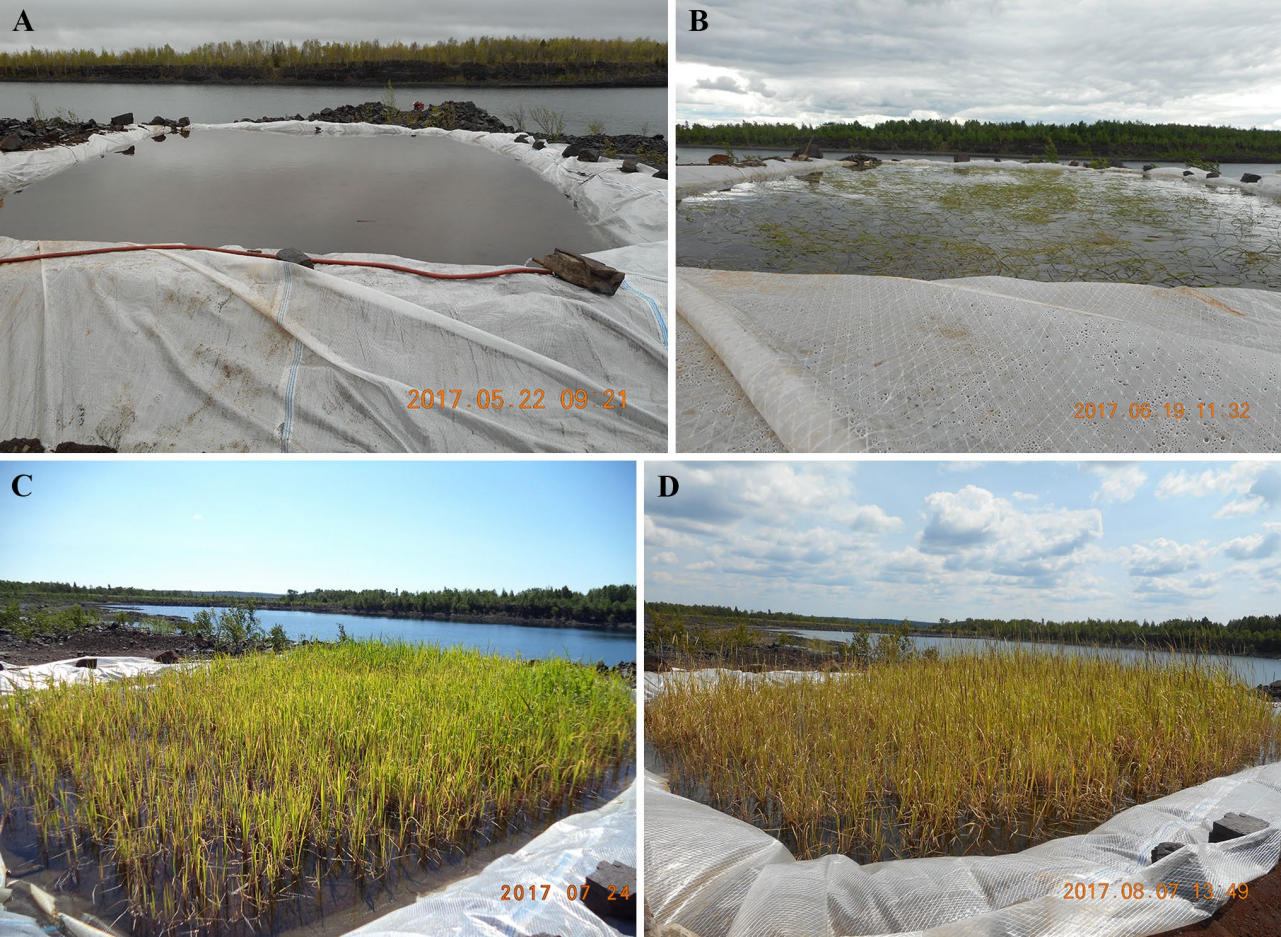
Paddy A. Images represent typical temporal WR phenological observations during 2017–2019 growing seasons: **A** seed distribution date and approximate yearly germination (2017-05-22); **B** floating leaf (2017-06-19); **C** aerial, flowering, seed production (2017-07-24); and **D** filled seeds throughout (2017-08-07). Yearly harvest occurred in August

**Fig. 2:**
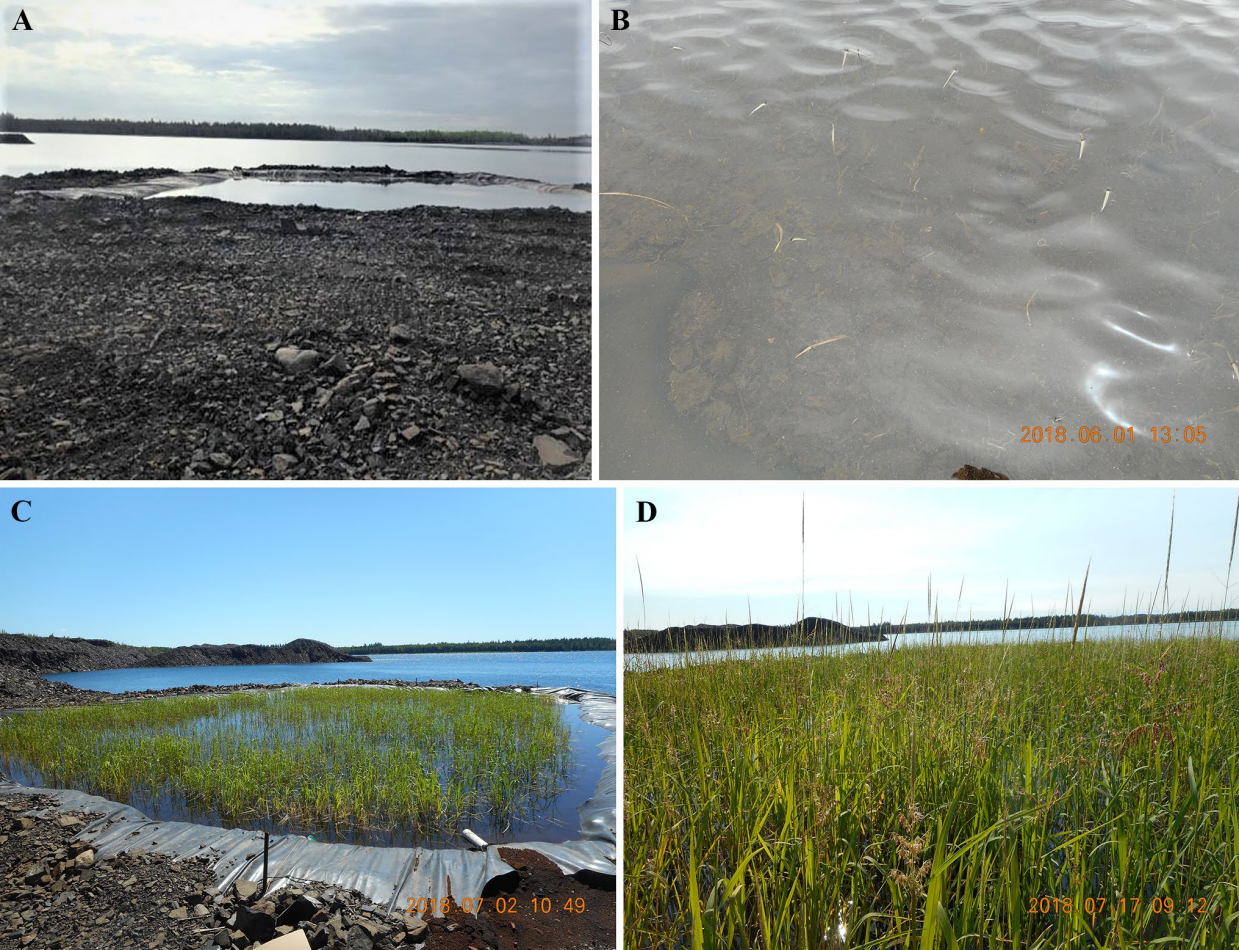
Paddy C. Images represent typical WR phenological observations during 2018 and 2019 growing seasons: **A** seed distribution date and approximate yearly germination (2018-05-22); **B** floating leaf (2018-06-01); **C** aerial (2018-07-02); and **D** flowering and seed production (2018-07-17). Yearly harvest occurred in August

Paddy A (initiated May 2017; ≈55 m^2^ surface area) and Paddy C (initiated May 2018; ≈150 m^2^ surface area) were constructed using onsite materials as impermeable perimeter berms, with an impermeable liner as the base. Average substrate and water depths at initial seed distribution were ≈25 and 25 cm in Paddy A, and 46 and 33 cm in Paddy C. Source seeds were harvested from lakes in northern MN, and were broadcast at a rate of ≈56 kg ha^−1^ (wet weight) in each paddy—approximately one Kg in Paddy A, and 2.5 kg in Paddy C. In an effort to ensure viable WR seed would be present in Paddy A substrate during winter months, additional seed was broadcast (0.45 kg; one lb.) throughout Paddy A during October 2017. As a result of observed spring 2018 WR germination in Paddy A, no additional WR seed was broadcast in either paddy—Paddy C only received WR seed in May 2018 for initial seeding. Paddies were visited approximately weekly during their respective growing seasons; Fig. 1 details typical Paddy A temporal observations during 2017–2019 growing seasons; Fig. 2 details typical Paddy C temporal observations during 2018–2019 growing seasons.

Unlike commercial WR paddies, neither of these paddies were drained at the end of, or between, growing seasons. Inflow water from their respective pits was maintained until freeze-up, and was re-initiated in late-March/early-April each year; no nutrients (fertilizer) were amended to substrate in either paddy. The planted area of each paddy was delineated into one-square-meter quadrats for randomized substrate, plant, and seed sampling purposes. Water was directly sampled from the inflow and outflow of each paddy once per growing season during harvest. Water samples were delivered to Pace Analytical Laboratories (Pace) in Virginia, MN. Substrate samples from the top 10 cm of each paddy were obtained from randomly selected quadrats using a 4.8 cm internal diameter sediment core sleeve. All substrate samples were frozen and delivered to the Lakehead University Environmental Laboratory (LUEL; Lakehead University, Thunder Bay, ON, Canada).

### Pore Water Sampling

Pore water (PW) characteristic samples were obtained approximately every 30 days from inflow, middle, and outflow areas within each paddy throughout each growing season. Two PW sampling methods were used during this study: diffusion-based peepers and Rhizons. At least 50 mL were required for H_2_S concentration determination. All PW samples were delivered to Pace. Field measurements of *p*H, dissolved oxygen, temperature, and specific conductance were obtained immediately prior to PW sampling from at least the inflow and outflow of each paddy using a calibrated YSI^®^ ProPlus^®^ or a Hach^®^ MS5 HydroLab^®^.

Diffusion-based peepers were 50 mL centrifuge tubes with a 0.45 µM pore-size filter sealed to the cap, filled (no headspace) with deoxygenated double-distilled water (Hesslein 1976; Teasdale et al. 1995; Azcue et al. 1996; LaForce et al. 2000; Jacobs 2002; Jorgenson 2013; Peijnenburg et al. 2014). These peepers were prepared and maintained under nitrogen headspace for ≈14 d prior to deployment; and transported to field sites in sealed nitrogen-purged and -filled bags. Rhizons are filter assemblies (0.12–0.18 µM pore-size) used for PW aspiration into the sample container protected from atmospheric exposure. All peepers and Rhizon assemblies were deployed at a 10 cm substrate depth. A mixture of sodium hydroxide and zinc acetate (C&G Containers) was used as the preservative for PW H_2_S samples.

Initially, Rhizons were attached to peepers with an aspiration tube connected and anchored on the paddy berm. Pore water was aspirated into the sample container, immediately prior to peeper retrieval. This avoided substrate disturbance prior to PW aspiration. New Rhizon assemblies were used for each peeper re-deployment. However, due to Rhizon filter pore size limitations, clogging was an observed problem, periodically resulting in lower than required sample volume. Due to filter clogging and subsequent periodic insufficient sample volumes, use of Rhizons was discontinued following 2018. Diffusion-based peepers were used to capture PW characteristics throughout the 2019 growing season.

### Plant and Seed Sampling

Groups of plants were sampled from randomly selected quadrats in Paddy A during 2018 and 2019, and the stem count was completed in a 0.1 m^2^ area inside each selected quadrat. Stem density in Paddy C was ≈1/10 of Paddy A; therefore, all individual stems were counted, and random plants were sampled, from randomly selected quadrats during 2018 and 2019. Following observation of filled seeds, plant and seed samples were harvested from randomly selected quadrats in both paddies typically on the same date. However, due to repeated extensive goose herbivory in Paddy C during 2019, plants were sampled on Aug. 01, and seeds were sampled on Aug. 23, 2019.

On July 10, 2018, during the early flowering stage, whole WR plants (including roots) were harvested from randomly selected quadrats within each paddy. Roots were rinsed in paddy surface water to remove substrate and other materials. Plants were stored refrigerated in Ziploc^®^ bags, transported to LUEL, and prepared for scanning electron microscope (SEM) and energy dispersive x-ray (EDX) characterization. SEMs were obtained from root surface areas visually suspect of (1) typical root tissue; and (2) iron-containing coatings [Fe (III) O-(OH) and FeS]. Points for EDX characterization were chosen based on visual appearance and SEM imagery of the WR root surface. SEM–EDX characterization was completed at Lakehead University Instrumentation Laboratory (LUIL; Lakehead Univ, Thunder Bay, ON, Canada) as described in Jorgenson et al. (2013).

### Laboratory Analytical Methods

Quality assurance/quality controls (QA/QC) for water samples characterized by Pace included reference standards, matrix spikes, and conformed to The National Environmental Laboratory Accreditation Conference (NELAC) Standards and the Pace Quality Assurance Manual. QA/QC field-sampling practices included collection of field blank and duplicate inflow and outflow water samples. Analytical methods for water and PW conformed to NELAC Standards and followed those described in Pace (2020a, b). Specifically, Al, B, Ca, Fe, Mg, Mn, and Na in water and Fe in PW were measured using ICP-OES (EPA 200.7); and Cu was measured using ICP-MS (EPA 200.8) following acidification to pH < 2.0 using trace metal grade HNO_3_. Chloride (Cl) and SO_4_ were separated from non-chemically-preserved samples through a series of ion-selective columns and measured using IC (method 300.0). Samples for sulfide (as H_2_S) were preserved using a sodium hydroxide and zinc acetate mixture (C&G Containers) and were measured according to Standard Method 4500-S2-G.

All substrate, plant, and seed sample analyses were completed at LUEL, a Canadian Association of Laboratory Accreditation (CALA) ISO 17025 accredited laboratory. Preparation and analytical procedures for substrate, plant, and seed samples reference Forest Canada, ASTM, American Soil and Plant Council, and/or USEPA methods; the LUEL Quality Assurance Manual; and followed those described in Lee and McNaughton (2004). All analyses followed standard operating procedures and included blank, quality control, and replicate samples. Substrate samples were collected using new 4.5 cm internal-diameter cellulose acetate butyrate sediment core sleeves. As a result of observed variability between substrate samples, laboratory (LUEL) replicate samples were used in place of field duplicate samples. Total nitrogen (when possible) was measured on dry substrate using a combustion ELVario cube carbon-hydrogen–nitrogen-sulphur analyzer. Loss on ignition (LOI) was measured by drying 20 mL of substrate at 80 °C, weighing, ashing at 600 °C, and re-weighing. Substrate pH was measured as a 1:1 substrate:deionized water mixture using a Mettler Toledo meter with an InLinePro pH sensor. Bulk density was measured by weighing 20 mL of wet substrate, drying at 80 °C, and re-weighing. Phosphate was determined using the BRAY P2 method—Al and Fe phosphates are dissolved in ammonium fluoride and measured colorimetrically using a SKALAR analyzer. Al, Cu, Fe, Mn, S, and Zn were extracted in 0.1 N trace metal grade HCl, while Ca, K, Mg, and Na were extracted in ammonium-acetate at pH 7.0; all of which were measured using ICP. Substrate samples were processed as wet samples to better represent field conditions. Measured analyte concentrations were corrected for bulk density. Plant, or seed, biomass (≥ 0.5 g) was digested using a CEM Mars Express microwave in Express Teflon closed vessels in one mL of HNO_3_ and three mL of trace metal grade HCl and diluted using 25 mL of deionized water. Total P, Al, Ca, Cu, Fe, Mg, Mn, Na, S, and Zn in this dilution were measured using a Varian ICP-AES. Total N was measured using a SKALAR analyzer following microwave digestion and concentration in trace metal grade H_2_SO_4_ catalyzed with a metal sulphate.

### Statistical Analyses

All surface water, substrate, WR plant and seed, and PW data were organized using Microsoft^®^ Excel^®^ or SigmaPlot-SigmaStat^®^ v14.0 (Systat Software, Inc.) for table and graphical representation. Statistical analyses and treatment of WR plant data were completed using SigmaPlot-SigmaStat^®^ v14.0. t-Tests or one-way analysis of variance (ANOVA) were used to compare two or more groups; specifically, WR stem density, stem height, shoot DWB, seed production, and seed DWB. Holm-Sidak multiple comparison was used to discern significant differences between three or more groups if data met assumptions of normal distribution and equal variance. Data were natural logarithmic transformed if normal distribution and/or equal variance assumptions were not met. If data transformation failed to correct non-normal distribution and/or variance inequality, raw data were used for statistical treatment, followed by ANOVA on ranks between three or more groups and Dunn’s multiple comparison to discern significant differences between groups. A Mann–Whitney Rank Sum test was used to discern significant differences between groups used for t-test comparisons when data were not corrected for non-normal distribution or variance inequality through natural logarithmic transformation. Non-transformed data were used for table and figure display purposes.

## Results

### Water, Substrate, and Pore Water Characteristics

Characteristics of Paddy A and C inflow and outflow waters remained similar between 2017–2019 growing seasons (Table 1; Supplemental Table S-1). In particular, aqueous SO_4_, calcium, and magnesium remained similar between and during growing seasons, and between inflow and outflow of each respective paddy. Concentrations of Al, Cd, Cu, Hg, and Pb in Pit A water have been at or below detection limit (< 1–5 µg L^−1^); in the case of Hg less than 1.0 ng L^−1^. Pit C water Hg concentrations have been < 2.0 ng L^−1^ (Table S-1). These concentrations are approximately three orders of magnitude less than those resulting in toxicity to WR (Lee and Hughes 1998; Supplemental Figs S-1 and S-2); and not likely to result in adverse WR responses. Measured substrate characteristics from Paddies A and C are listed in Table 2. General decreases in plant nutrient elements were observed in each paddy; in particular, ammonium decreased six- and twelve- fold in Paddy A and C substrate, respectively, since initiation. Variability was observed between diffusion-based peepers and Rhizon H_2_S concentrations in both paddies. Average Paddy A and C peeper PW characteristics are listed in Table 3 (Paddy A and C peeper and Rhizon data available in Supplemental Tables S-2–4 and 5–6, respectively). Average Paddy A H_2_S concentrations ranged from < 0.078–1.58 mg L^−1^ in peeper samples. Average Paddy C PW H_2_S concentrations ranged from 0.337 to 2.528 mg L^−1^ in peeper samples. Notably, H_2_S exceeded the suggested 0.165 mg L^−1^ protective level (MPCA March 2015) for WR in nearly all PW samples by several fold.

**Table 1:** Paddy A and C inflow and outflow characteristics

	Paddy A						Paddy C			
	Inflow			Outflow			Inflow		Outflow	
	2017 (n = 9)	2018 (n = 5)	2019 (n = 6)	2017 (n = 9)	2018 (n = 5)	2019 (n = 6)	2018 (n = 5)	2019 (n = 6)	2018 (n = 5)	2019 (n = 6)
*p*H (SU)	8.3 ± 0.2	8.4 ± 0.1	8.2 ± 0.2	8.5 ± 0.2	8.0 ± 0.2	8.0 ± 0.1	8.4 ± 0.1	8.4 ± 0.1	8.4 ± 0.1	8.3 ± 0.1
Cond. (µS cm^−1^)	1235 ± 149	1202 ± 24	1157 ± 26	1252 ± 154	1195 ± 28	1133 ± 77	2593 ± 100	2585 ± 128	2543 ± 146	2576 ± 139
Alk. (mg L^−1^)	329	288	354	328	310	364	461	554	487	556
Ca	50	47	43	49	46	40	29	31	32	32
Mg	134	126	119	131	130	121	383	369	405	380
Na	32	28	25	32	30	25	53	49	54	50
SO_4_	403	368	359	405	370	360	1,310	1,270	1,340	1,290
Cu (µg L^−1^)	NM	1.8	BD	NM	1.3	BD	2.1	BD	2.3	1.1
Mn	NM	25	32	NM	42	14	20	25	79	68

*p*H and conductance measured monthly (avg ± one SD). Alk. through Mn measured at season harvest. Units for Alk. through SO_4_ are mg L^−1^; Cu and Mn are µg L^−1^ (*n* = 1 for each inflow-outflow sampling event per location)

*NM* not measured, *BD* below detection limit

**Table 2:** Substrate characteristics during WR harvest events (avg ± one SD). Units for NH_3_ through Na are µg g^−1^

	Source (n = 1)	Paddy A			Paddy C	
		2017 (n = 3)	2018 (n = 3)	2019 (n = 11)	2018 (n = 3)	2019 (n = 8)
Total N (%)	NM	0.2 ± 0.1	0.2 ± NC	0.4 ± 0.2	0.1 ± NC	0.8 ± 0.3
LOI	12.3	8.2 ± 3.8	NM	16.5 ± 6.2	5.0 ± 1.1	27 ± 10
*p*H (SU)	6.6	5.8 ± 0.1	7.6 ± 0.1	7.0 ± 0.2	7.4 ± 0.1	6.9 ± 0.1
Bulk Density (g cm^−3^)	0.4	0.3 ± 0.1	0.5 ± 0.1	0.6 ± 0.1	0.2 ± NC	0.3 ± 0.1
NH_3_ + NH_4_-N (µg g^−1^)	24.8	14.7 ± 5.4	18.1 ± 4.9	3.4 ± 1.2	6.2 ± 1.2	1.5 ± 0.5
PO_4_	5.1	2.0 ± 0.5	6.8 ± 2.6	1.5 ± 0.7	0.7 ± 0.1	0.6 ± 0.3
Al	357	149 ± 31	382 ± 26	112 ± 43	86 ± 5	39 ± 17
Cu	2.1	1.0 ± 0.2	3.1 ± 0.3	0.4 ± 0.4	0.7 ± 0.1	0.1 ± 0.1
Fe	1,819	296 ± 45	1013 ± 106	2,181 ± 856	222 ± 5	803 ± 217
Mn	267	47 ± 22	75 ± 17	40 ± 31	27 ± 1	12 ± 7
S	214	115 ± 28	178 ± 9	240 ± 216	175 ± 26	97 ± 62
Zn	7.0	2.1 ± 0.3	5.3 ± 0.7	1.8 ± 0.9	1.0 ± 0.1	0.4 ± 0.2
Ca	3,235	1,031 ± 421	1468 ± 277	766 ± 225	271 ± 48	172 ± 72
K	86	37 ± 6	59 ± 23	21 ± 7	11 ± 3	6 ± 3
Mg	1,246	573 ± 202	716 ± 136	294 ± 80	359 ± 61	156 ± 52
Na	23	42 ± 18	59 ± 12	24 ± 6	29 ± 3	11 ± 4

*NM* not measured, *NC* not calculable

**Table 3:** Paddy A and C peeper H_2_S data (mg L^−1^; avg ± one SD; n = 3 for each sampling event at each location)

Paddy A		Paddy C	
Jul. 31, 2017	BD	NM	NM
Aug. 31	0.421 ± 0.122	NM	NM
Sept. 29	0.715 ± 0.245	NM	NM
Oct. 27	0.427 ± 0.193	NM	NM
Jun. 01, 2018	0.287 ± 0.051	NM	NM
Jul. 02	0.929 ± 0.682	Jul. 02, 2018	0.485 ± 0.190
Aug. 02	0.484 ± 0.332	Aug. 02	0.337 ± 0.049
Aug. 30	0.773 ± 0.202	Aug. 30	0.402 ± 0.069
Oct. 01	1.05 ± 0.086	Oct. 01	0.422 ± 0.079
Jun. 04, 2019	1.14 ± 1.094	Jun. 04, 2019	0.903 ± 0.585
Jul. 02	0.469 ± 0.182	Jul. 02	0.562 ± 0.132
Aug. 01	1.50 ± 1.091	Aug. 01	2.528 ± 2.477
Sept. 03	1.58 ± 0.748	Sept. 03	0.565 ± 0.098
Oct. 03	0.896 ± 0.462	Oct. 03	0.930 ± 0.120

*BD* below detection limit (0.078 mg L^−1^), *NM* not measured

### Wild Rice Phenology and Productivity

Wild rice phenological development was similar between 2017–2019 growing seasons for Paddy A (Fig. 1) and 2018–2019 growing seasons for Paddy C (Fig. 2). Dates on which specific WR phenological stages were observed in Paddies A and C are listed in Tables 4, 5, respectively. Plant and seed harvest dates for each paddy were typically the same date during each growing season, and within the same two weeks of August between growing seasons indicating no identifiable adverse influences on WR temporal phenological development within or between growing seasons from Pit A or C water-associated exposures under these conditions.

**Table 4:**
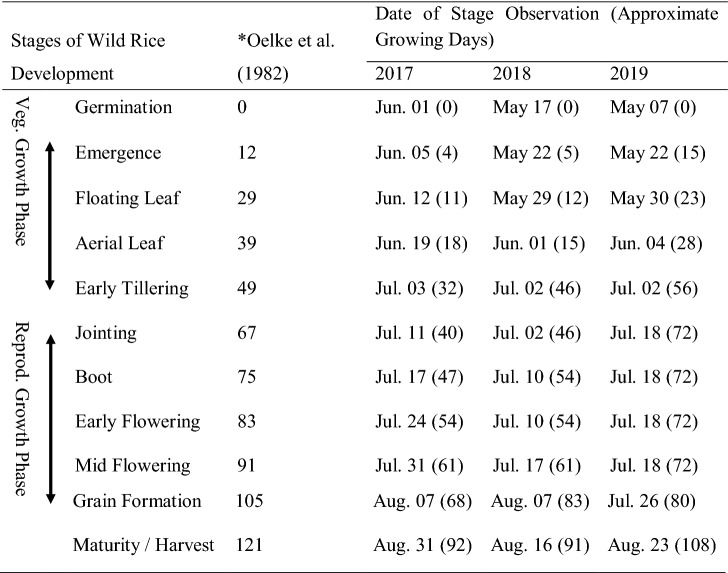
Paddy A wild rice phenology as observed during 2017–2019 growing seasons. Phenological development stages as used by Oelke et al. (1982)

*Days from germination; from Oelke et al. (1982) – WR plant development (K2 variety) Aitkin County, MN, USA

**Table 5:**
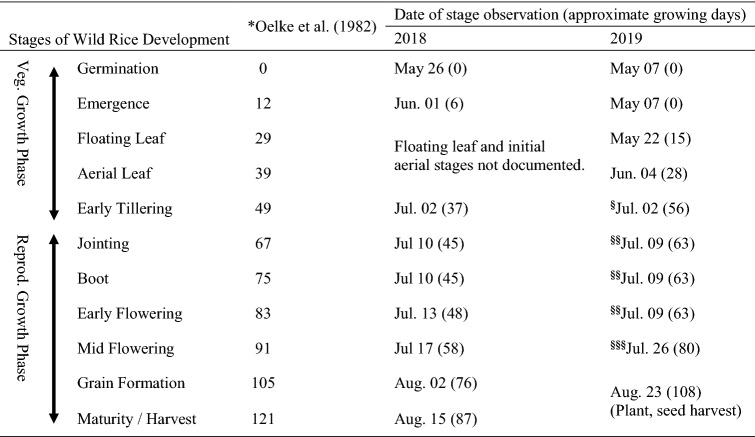
Paddy C wild rice phenology as observed during 2018 and 2019 growing seasons. Phenological development stages as used by Oelke et al. (1982)

*Days from germination; from Oelke et al. (1982)—WR plant development (K2 variety) Aitkin County, MN, USA

^**§**^Goose herbivory first observed

^**§§**^Extensive goose herbivory; first flowering observed

^**§§§**^Additional, extensive goose herbivory observed; early seed formation observed on some remaining WR

**Fig. 3:**
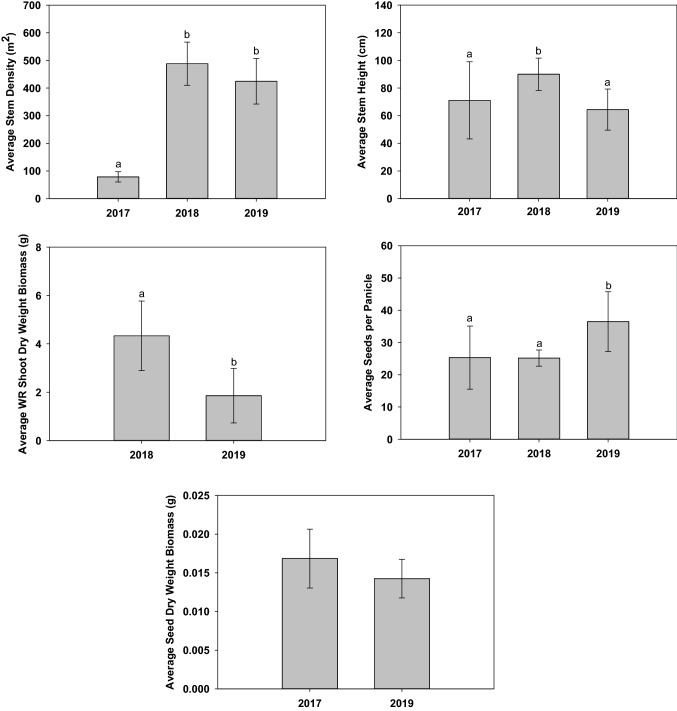
Paddy A average stem density, stem height, shoot dry weight biomass, filled seeds per panicle, and seed dry weight biomass. No plant samples were obtained during 2017. All seeds harvested during 2018 were lost due to a freezer failure. Error bars represent one standard deviation. Differing letters (a and b) indicate statistical significance at alpha 0.05

One-way ANOVAs were used to discern differences between stem density, stem height, shoot DWB, seeds per panicle, and seed DWB in Paddy A (Fig. 3). Wild rice stem density throughout the paddy differed significantly between growing seasons (F_(2,40)_ = 365.362 *p* < 0.001). Specifically, stem density increased by ≈10 × between 2017 and 2018, and remained at this density through 2019. Paired comparison tests indicated that, the average 2019 stem density was statistically higher than in 2017 (*p* < 0.001), but was not significantly different from 2018 (*p* = 0.106). Average 2018 stem heights were significantly higher (F_(2,68)_ = 8.560 *p* < 0.001) than both 2017 (*p* = 0.016) and 2019 (*p* =  < 0.001), with a concurrent statistical decrease in 2019 shoot DWB (t_(21)_ = 4.568 *p* < 0.001). No statistical difference (F_(2,26)_ = 5.615 *p* = 0.010) was identified between average seeds per panicle between 2017 and 2018 (*p* = 1.000), despite the ≈10 × increase in stem density. However, paired comparison tests indicated that the average number of seeds per panicle during 2019 was significantly higher than in 2017 (*p* = 0.046) and 2018 (*p* = 0.030). t-Tests indicated no statistical difference between seed DWB between 2017 and 2019 growing seasons (t_(13)_ = 1.467 *p* = 0.166).

**Fig. 4:**
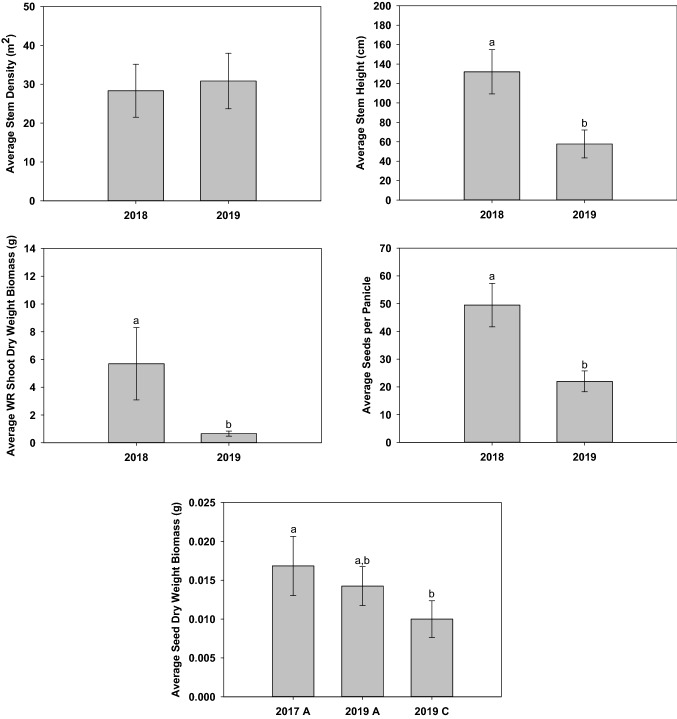
Paddy C average stem density, stem height, shoot dry weight biomass, filled seeds per panicle, and seed dry weight biomass. All seeds harvested during 2018 were lost due to a freezer failure. Error bars represent one standard deviation. Differing letters (a and b) indicate statistical significance at alpha 0.05

T-tests were used to discern differences between WR stem density, stem height, shoot DWB, seeds per panicle, and seed DWB in Paddy C (Fig. 4). On July 09, 2019, goose herbivory was observed throughout the paddy. Also documented on this date were initial observations of flowering; male flowers were present on several plants throughout this paddy. Despite extensive and repeated goose herbivory, t-test indicated no statistical difference between average 2018 and 2019 stem density (t_(22)_ = − 0.877 *p* = 0.390). However, the 2019 average stem height was significantly less than in 2018 (t_(34)_ = 11.995 *p* < 0.001). Additionally, the average WR shoot DWB (t_(19)_ = 16.971 *p* < 0.001) and seeds per panicle (t_(16)_ = 8.060 *p* < 0.001) were significantly less than in 2018. Due to a freezer failure in 2018, all harvested seeds were lost. Therefore, average 2019 Paddy C seed DWB was contrasted to average 2017 and 2019 Paddy A seed DWB. ANOVA indicated that the average 2019 Paddy C seed DWB was significantly less than the average 2017 Paddy A seed DWB (F_(2,20)_ = 8.679 *p* = 0.002). Significantly decreased stem height, seed production, shoot DWB, and seed DWB during 2019 is likely resultant from extensive and repeated goose herbivory.

**Fig. 5:**
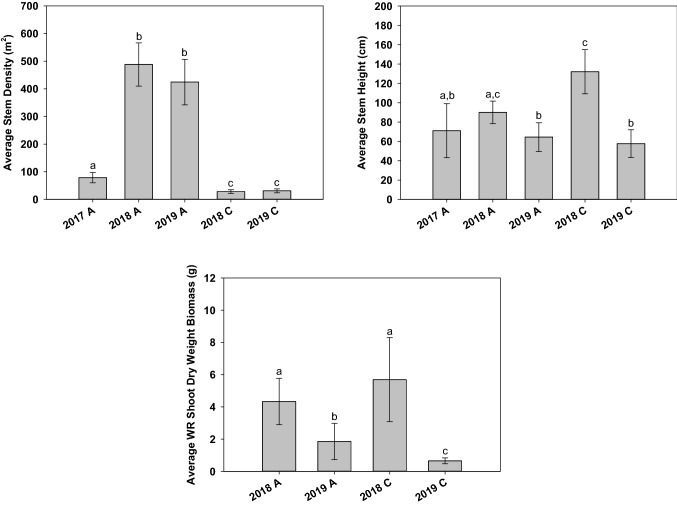
Comparison between average Paddy A and C stem density, stem height, and shoot dry weight biomass. Error bars represent one standard deviation. Differing letters (a, b, c) indicate statistical significance at alpha 0.05

One-way ANOVAs were used to discern differences between Paddy A and Paddy C WR stem density, stem height, shoot DWB (Fig. 5). Average stem density in Paddy A (2017, 2018, 2019) was statistically higher (F_4,60_ = 451; *p* < 0.001) than Paddy C in 2018 (*p* < 0.001) and 2019 (*p* < 0.001) in all growing seasons. With the exception of Paddy A (2018), average stem height in Paddy C (2018) was statistically higher than other growing seasons (*p* < 0.001). Despite higher stem height, average Paddy C (2018) shoot DWB was not significantly different from Paddy A (2018) (*p* = 0.636), but was statistically higher than other growing seasons (*p* < 0.001).

### Plant and Seed Characteristics

Chemical characteristics of WR plant and seed samples from both paddies are listed in Table 6. With the exception of Ca and Zn, which were higher in plants harvested in 2019 than 2018, chemical characteristics of WR between growing seasons in Paddy A were similar; specifically, total plant N averaged 0.74% (± 0.10) for each season. Shoots of plants harvested in 2019 from Paddy C contained higher concentrations of N, P, and S than in 2018, potentially due to nutrient uptake into plant tissues and internodal re-growth following herbivory prior to plant harvest. Regardless of surface water, substrate, PW, or plant tissue characteristics, seed characteristics were similar between growing seasons. Percent N and S content of seeds harvested from Paddy A during 2017 averaged 1.24 (± 0.12) and 0.12 (± 0.013), respectively. The average percent N content of seeds harvested in 2019 significantly increased to 1.55 (± 0.05) (t_(13)_ = − 5.411; *p* = 0.0002); the average percent S content remained at 0.12 (± 0.004). During 2019, t-tests indicated that seeds harvested from Paddy C contained significantly higher concentrations of N (t_(10)_ = − 4.116 *p* = 0.002) and S (t_(10)_ = − 3.449 *p* = 0.006) when contrasted to seeds harvested from Paddy A. No significant difference was identified between concentrations of P (t_(10)_ = − 0.175 *p* = 0.865) or K (t_(10)_ = 0.376 *p* = 0.715) in Paddy C seeds contrasted to Paddy A seeds. Despite goose herbivory in Paddy C, harvested plants appeared generally healthy (no observations of nutrient deficiencies or WR diseases), seeds had been filled, and overall WR did not appear to have been adversely influenced by chemical characteristics of Pit C water or water-associated exposures under these conditions.

**Table 6:** Characteristics of shoot and seed tissue (avg ± one SD). Units for total N through Fe are %; Al through Zn are µg g^−1^

	*Rat River Bay		Paddy A				Paddy C		
	Shoots	Seeds	Shoots		Seeds		Shoots		Seeds
	2018 (n = 3)	2018 (n = 24)	2018 (n = 12)	2019 (n = 11)	2017 (n = 9)	2019 (n = 6)	2018 (*n* = 12)	2019 (n = 9)	2019 (n = 6)
Total N (%)	0.83 ± 0.04	1.19 ± 0.12	0.68 ± 0.08	0.75 ± 0.09	1.24 ± 0.12	1.55 ± 0.090	0.74 ± 0.34	1.89 ± 0.31	1.86 ± 0.10
P	0.10 ± 0.01	0.32 ± 0.02	0.13 ± 0.02	0.08 ± 0.02	NM	0.243 ± 0.028	0.10 ± 0.03	0.25 ± 0.16	0.25 ± 0.03
K	0.49 ± 0.07	0.42 ± 0.08	1.50 ± 0.23	1.18 ± 0.39	NM	0.361 ± 0.024	2.37 ± 0.49	2.18 ± 0.42	0.35 ± 0.05
S	0.09 ± 0.01	0.11 ± 0.01	0.30 ± 0.07	0.46 ± 0.1	0.12 ± 0.01	0.118 ± 0.010	0.31 ± 0.06	0.90 ± 0.17	0.14 ± 0.01
Ca	1.14 ± 0.05	0.02 ± 0.01	0.59 ± 0.32	1.73 ± 0.81	NM	0.038 ± 0.006	0.16 ± 0.03	0.32 ± 0.22	0.04 ± 0.01
Mg	0.23 ± 0.03	0.10 ± 0.01	0.36 ± 0.05	0.37 ± 0.03	NM	0.102 ± 0.008	0.43 ± 0.09	0.78 ± 0.13	0.12 ± 0.02
Na	0.60 ± 0.06	0.01 ± 0.01	0.50 ± 0.08	0.48 ± 0.10	NM	0.004 ± 0.001	0.42 ± 0.11	0.67 ± NC	0.01 ± 0.01
Fe	0.12 ± 0.02	0.01 ± 0.01	0.10 ± 0.04	0.32 ± 0.20	NM	0.020 ± 0.003	0.10 ± 0.02	0.11 ± 0.08	0.01 ± 0.01
Al (µg g^−1^)	804 ± 136	22 ± 35	80 ± 37	248 ± 314	NM	7 ± 3	235 ± 57	172 ± 146	6 ± 1
Cu	19 ± 15	5 ± 2	1.5 ± 0.3	2.2 ± 0.7	NM	4.4 ± 0.8	1.4 ± 0.4	2.4 ± 1.1	4.2 ± 0.9
Mn	1434 ± 122	25 ± 7	1199 ± 406	586 ± 256	NM	33 ± 7	333 ± 81	1664 ± 869	37 ± 12
Zn	21 ± 3	32 ± 8	9.6 ± 1.2	22 ± 11	NM	25 ± 2	8.3 ± 2.2	12.1 ± 5.9	11.5 ± 2.7

*Non-mining/-SO_4_ influenced site; data fromTedrow and Lee (2021)

*NM* not measured

### SEM–EDX WR Root-Surface Characterization

Surfaces of WR roots harvested during July 2018 were characterized by SEM and EDX as described by Jorgenson et al. (2013). Points chosen for EDX characterization were selected based on visual appearances: white appearance may be more indicative of typical root tissue; orange appearance may be more indicative of Fe (III) oxide/hydroxide coating. Neither Paddy A nor Paddy C plants contained identifiable root coatings indicative of FeS (supplemental Figs S-3 and S-4). However, visual appearance of most roots on WR plants was likely indicative of iron (III) oxy / hydroxide coatings. On Paddy A plants, C, O, and Ca were dominant elements in areas suspect of typical root tissue; and C, O, and Fe were dominant elements in areas suspect of Fe (III) oxide/hydroxide coatings. On Paddy C plants, C, O, S, and at times K, were dominant elements in areas suspect of typical root tissue; and C, O, and Fe (at times, multi-peak) were dominant elements in areas suspect of Fe (III) oxide/hydroxide root coatings. Despite aqueous SO_4_, PW H_2_S, and dissolved PW Fe concentrations all theoretically conducive to FeS complexation, neither visual appearance nor EDX-characterization of root coatings were indicative of FeS. However, during and following WR plant and seed harvest events, black coatings on roots were observed in both paddies. These coatings closely resemble FeS root coatings inferred and described by LaFond-Hudson et al. (2018, 2020) near/at growing season conclusion. Their observations and concluded rationale for FeS root coatings tend to represent phenological life stages of WR—near seed abscission from pedicels, root radial oxygen loss (ROL) ceases allowing for conditions more favorable to FeS complexation to occur. Additional surface characterization of black-appearing WR roots in the current study was not pursued due to the similarity with those described by LaFond-Hudson et al. (2018, 2020) as likely FeS. Throughout growing seasons, WR plants appeared healthy, were productive, and observations of Fe-containing root coatings appeared more representative of WR phenological stage and root ROL than overlying water, pore water, or substrate characteristics.

## Discussion

Primary concerns influencing use of mining-influenced waters for irrigation are sufficiently high concentrations of potentially toxic elements and SO_4_ concentrations. Mining-influenced waters used for the current study did not contain sufficiently high concentrations of elements such as As, Cu, Ni, Fe, Pb, Cd, Hg, and/or Zn to be considered potentially problematic (Lee and Hughes 1998). However, aqueous SO_4_ concentrations (Table 2) were higher than 1) the MN SO_4_ water quality criterion for WR (10 mg L^−1^), and 2) concentrations suggested to be problematic for WR in general (Myrbo et al. 2017; Pastor et al. 2017; LaFond-Hudson et al. 2018). Since no adverse responses from WR were observed in the current study, the overall focus shifted to plant and seed characteristics and substrate nutrient availabilities. Tedrow and Lee (2021) successfully grew WR to maturity in mining- and non-mining- influenced lake sediments. Time to maturity was the same for all plants but height and DWB were lower for plants grown in mining sediments. The size difference was attributed to lower concentrations of ammonium in mining-influenced sediments than the reference sediment. Seed chemical characteristics between all exposures were similar and not necessarily reflective of respective sediment characteristics. Physical plant characteristics such as overall size and productivity were primary differences between these sediment exposures, more likely a result of multi-fold lower concentrations of ammonium in these mining-influenced sediments than the reference sediment.

### Wild Rice Responses

In previous microcosm and bucket-style studies, WR tended to grow more slowly, decrease in overall size, be less productive, and generally fail to thrive specifically in exposures of 300 mg SO_4_ L^−1^ (Pastor et al. 2017; LaFond-Hudson et al. 2018). In the current study, SO_4_ exposure concentrations exceeded these previous studies by ≈50 mg L^−1^ (Pit A water) and 1,050 mg L^−1^ (Pit C water). Hydrogen sulfide is known to be toxic to aquatic plants in concentrations ranging from 0.4–11.0 mg L^−1^ (Armstrong and Armstrong 2005; Lamers et al. 2013). In both paddies, concentrations of pore water H_2_S routinely exceeded the MPCA-suggested 0.165 mg L^−1^ (MPCA 2015) protective level by multiple-fold. Despite these exposures, no adverse WR responses were observed (decreased size and/or productivity; delayed development) that could be attributable to SO_4_- or H_2_S- specific exposures.

In October 2017, an additional 0.45 kg of WR seed was amended to Paddy A to ensure viable WR would be present immediately prior to freeze-up and would have the chance to overwinter in Paddy A. WR stem density increased ≈10 × between 2017 and 2018/2019 in Paddy A. WR stem density in Paddy C ranged from 18 to 41 stems m^−2^ and did not statistically differ between 2018 and 2019; and was 50–90% lower than Paddy A stem density in 2017–2019. Paddy A germination in Spring 2018 verified viable WR seed could overwinter in Pit A water; therefore, no additional WR seed was amended to Paddy C. In the absence of a significant stem density decrease in either paddy between growing seasons, SO_4_ does not appear to be detrimental for WR germination. Increased stem density in Paddy A is more likely a result of viable seed amendment in October 2017.

### Wild Rice Development

In addition to concerns about adverse influences from SO_4_ on WR distribution, density, and productivity, specific developmental delays have been documented. LaFond-Hudson et al. (2018, 2020) concluded that an observed decrease in developmental rate(s) beginning during vegetative growth phase, and promulgated through reproductive maturity, was in response to exposures of 300 mg SO_4_ L^−1^. Although the observed delay was only a few days, the difference was significant between specific treatment groups. In the current study, although differences existed between dates of phenological stages observation, this difference cannot be attributed to SO_4_ exposure alone. Time between paddy inspections and subjective assessments of phenological development stages (Oelke et al. 1982; Sims et al. 2012a, b) are also potential reasons for observed temporal development differences; and must be ruled-out as causative prior to considering SO_4_ as the reason for developmental delay. Phenological development of WR in both paddies followed typical temporal patterns (Tables 4, 5).

### Plant and Seed Tissue Chemistry

Nutrient element concentrations of WR plant tissue have been reported by Hildebrandt et al. (2012), with total N (%) in shoot tissue reported as 0.85. In the current study, Paddy A WR shoot N (%) was 0.68 and 0.75 in 2018 and 2019; and Paddy C WR shoot N (%) was 0.74 and 1.89 in 2018 and 2019, respectively. The higher Paddy C WR % plant tissue N in 2019 may be attributed to N uptake and internodal re-growth, a response to repeated goose herbivory throughout the paddy (Weir and Dale 1960). Following observation of goose herbivory, netting was installed around Paddy C to exclude waterfowl. LaFond-Hudson et al. (2020) reported WR seed N (%) content of 1.89 for non-SO_4_ exposed WR, and 2.28 for SO_4_ exposed (300 mg L^−1^) WR. In the current study, seeds harvested from Paddy A WR were 1.24 and 1.55% N in 2017 and 2019, respectively. Seeds harvested from Paddy C WR contained 1.86% N in 2019. These values are close to the N content of non-SO_4_ amended WR reported by LaFond-Hudson et al. (2020). Additionally, average seed DWB was similar between non-SO_4_ amended WR (15.26 mg; LaFond-Hudson et al. 2020) and Paddy A WR (15.00 mg). These data indicate a lack of measurable influence of aqueous SO_4_ in determining specific seed characteristics of WR grown in Pit A and C waters.

### Nutrient Limitations

As detailed in Day and Lee (1989), Lee (2002), Lee and McNaughton (2004), Oelke (1982, 1997), and Sims (2012a, b), sufficiently high nutrients such as ammonium and P are of critical importance to WR growth, development, and distribution; specifically, during early- and mid- season phenology. In the current study, a general decrease in substrate ammonium has been observed in each paddy between growing seasons. In the absence of other adversely influential factors, we can likely expect WR plants to decrease in density, biomass, productivity, or a combination of these and other characteristics as a result of decreased and decreasing ammonium bioavailability. This effect from N depletion on WR productivity was documented by Keenan and Lee (1988). In their study, after five years of intensive cultivation of WR in a northwestern Ontario lake, % N levels in the sediment had decreased from 1.5 to 0.2 with corresponding declines in WR productivity. Only after the lake remained fallow for two years was commercial WR production again feasible. A similar ammonium trend may be developing in paddies used in the current study. Walker et al. (2006, 2010) documented N sequestration in accumulated WR plant and root litter, concluding that decreases in WR density, distribution, and productivity may be attributed to N limitation as a result of litter sequestration. In the current study, this may be a developing condition based on decreased substrate ammonium and observed WR plant litter in each paddy. Inflow water *p*H (≈8.3) to each paddy is sufficiently high to decrease some elemental nutrient bioavailabilities such as Fe and Cu. However, this may be unlikely due to substrate pH typically slightly acidic to circumneutral (5.8–7.6) Therefore, the potential for decreased WR density, distribution, and productivity exists resulting from bioavailable N limitation and/or deficiency.

## Conclusions

The current study provided critical support for potential use of mining-influenced waters for WR irrigation. Throughout multiple consecutive growing seasons, WR was grown in paddy-scale in-situ exposures of mining-influenced waters of substantially different chemical characteristics. In this current study, WR did not adversely respond to these exposures, and in both paddies developed and produced viable seed. Based on data and observations obtained during this study, overlying water characteristics such as aqueous SO_4_ appeared to play a less important role in WR phenology, distribution, and productivity, than previously suggested. In the current study, any differences between multiple measured WR characteristics were associated with substrate nutrient availability. Additionally, no adverse effects from H_2_S were observed; and observed increases in S in Paddy A were correlated to increases in Fe. If additional significant adverse WR responses are observed, elements becoming more concentrated in paddy substrate will be investigated as potential sources of those responses.

Development and use of these paddy-scale bioassays allowed more accurate and field-relevant predictions of responses of WR to exposures of mining-influenced waters with elevated SO_4_. Continued research will focus on substrate nutrient-element amendment in these paddies to help verify or refute potential adverse WR influences due to substrate nutrient depletion. These data would help to inform water use decisions less focused on characteristics of overlying water and more focused on substrate nutrient bioavailability. More broadly, the current study provides some refutation of the concern specific to SO_4_ adversely influencing WR phenology in general. Research on Fe-H_2_S toxicity mitigation and use of WR for phytoremediation of mine influenced water are important and relevant subjects that would be best answered by the paddy approach used in this study. Further study is also required to discern influences on WR from water depth increases, herbivory, and other organisms such as *Apamea apamiformis* (wild rice worm) within these paddies.

## Supplementary Information

Below is the link to the electronic supplementary material.

Supplementary file1 (DOCX 2645 KB)
