# Supplementary material for: Use of Wild Rice (Zizania palustris L.) in Paddy-Scale Bioassays for Assessing Potential Use of Mining-Influenced Water for Irrigation
Source: Mine Water Environ. 2022 Nov 18;41(4):938–53. doi: 10.1007/s10230-022-00908-0 (PMC9741574; doi:10.1007/s10230-022-00908-0)
Supplement: Supplementary file 1 — Supplementary file1 (DOCX 2645 KB) [file 10230_2022_908_MOESM1_ESM.docx]

**S.I. Fig 1** Growth of WR seedlings after exposure to Pb for fourteen days (Pb-1, 0.0; Pb-2, 0.001; Pb-3, 0.01; Pb-4, 0.1; Pb-5, 1.0; and Pb-6, 10.0 mg L^-1^) (From Lee and Hughes 1998)

**S.I. Fig 2** Shoot and root areas of WR seedlings exposed to Pb for fourteen days (From Lee and Hughes 1998)

**S.I. Fig 3** Root SEM-EDX characterization from Paddy A WR (July 2018). **TOP:** EDX spectrum from ‘Spectrum 2’ location identified on SEM image representative of typical root tissue EDX spectra, with primary elements (L-R) C, O, Ca, and Fe. **Bottom:** EDX spectrum from ‘Spectrum 4’ location identified on SEM image representative of Fe (III)O-(OH) coated-appearing areas, with primary elements (L-R) C, O, Fe (multi-peak)

**S.I. Fig 4** Root SEM-EDX characterization from Paddy C WR (July 2018). **Top:** EDX spectrum from ‘Spectrum 1’ location identified on SEM image representative of typical root tissue EDX spectra, with primary elements (L-R) C, O, S, K, and Fe. **Bottom:** EDX spectrum from ‘Spectrum 2’ location identified on SEM image representative of Fe (III)O-(OH) coating appearing areas, with primary elements (L-R) C, O, and Fe (multi-peak)

**S.I. Table 1** Pit A and C surface water samples obtained for non-WR paddy project purposes during May and August 2017, 2018, and 2019 (Pit A water) and 2018 and 2019 (Pit C water) (n = 1 for each sampling event)

|  | Pit A | | | | | | Pit C | | | |
| --- | --- | --- | --- | --- | --- | --- | --- | --- | --- | --- |
|  | 2017 | | 2018 | | 2019 | | 2018 | | 2019 | |
|  | May | August | May | August | May | August | May | August | May | August |
| *p*H (SU) | 8.3 | 8.6 | 8.2 | 8.5 | 7.8 | 8.3 | 8.3 | 8.4 | 8.3 | 8.3 |
| Cond. (µS cm^-1^) | 1,299 | 1,274 | 1,217 | 1,200 | 1,173 | 1,175 | 2,442 | 2,591 | 2,533 | 2,508 |
| *Alk. (mg L^-1^) | 289 | 273 | 384 | 362 | 396 | 366 | 596 | 578 | 619 | 560 |
| Ca | 58 | 57 | 55 | 47 | 55 | 52 | 47 | 30 | 47 | 34 |
| Mg | 146 | 146 | 136 | 122 | 134 | 130 | 381 | 386 | 407 | 398 |
| Na | NM | NM | NM | NM | NM | NM | NM | NM | NM | NM |
| SO_4_ | 411 | 408 | 395 | 371 | 354 | 363 | 1,210 | 1,280 | 1,240 | 1,270 |
| Cu (µg L^-1^) | NM | NM | NM | NM | NM | NM | NM | NM | NM | NM |
| Mn | NM | NM | NM | NM | NM | NM | NM | NM | NM | NM |

NM = not measured

**S.I. Table 2** Paddy A H_2_S and SO_4_ in diffusion-based peeper PW samples (all mg L^-1^; n = 1 for each sampling event per location)

|  | H_2_S | | | SO_4_ | | |
| --- | --- | --- | --- | --- | --- | --- |
|  | Inflow | Middle | Outflow | Inflow | Middle | Outflow |
| Jul. 31, 2017 | BD | BD | BD | 205 | 152 | 83 |
| Aug. 31 | BD | 0.542 | 0.299 | 269 | 112 | 127 |
| *Sept. 29 | 0.794 | 0.971 | 0.379 | 17 | 54 | 28 |
| *Oct. 27 | 0.358 | 0.232 | 0.690 | 50 | 104 | 15 |

*H_2_S in Rhizon pore water samples on these dates were below detection; BD = below detection (< 0.078 mg L^-1^)

**S.I. Table 3** Paddy A H_2_S, dissolved iron (Fe), and SO_4_ in diffusion-based peeper, and H_2_S in Rhizon (Rh), PW samples (all mg L^-1^; n = 1 for each sampling event per location)

|  | Inflow | | | | Middle | | | | Outflow | | | |
| --- | --- | --- | --- | --- | --- | --- | --- | --- | --- | --- | --- | --- |
|  | Peeper | Rh. | *Fe | SO_4_ | Peeper | Rh. | *Fe | SO_4_ | Peeper | Rh. | *Fe | SO_4_ |
| Jun. 01, 2018 | 0.244 | 0.172 | 0.33 | 73 | 0.258 | 0.315 | NM | 43 | 0.358 | 0.665 | NM | 211 |
| Jul. 02 | 0.384 | 0.199 | 13.4 | 12 | 0.513 | **0.135** | 15.1 | 109 | 1.89 | 2.09 | 6.09 | 287 |
| Aug. 02 | **0.161** | 1.64 | 1.17 | 58 | 0.940 | 1.12 | 9.84 | 60 | 0.350 | 1.13 | 1.82 | 13 |
| Aug. 30 | 0.696 | 1.25 | NM | NM | 1.05 | 3.07 | NM | NM | 0.574 | 1.96 | NM | NM |
| Oct. 01 | 1.15 | **0.154** | 3.78 | 15 | 1.06 | 0.964 | 0.73 | 28 | 0.941 | 0.433 | 1.72 | 43 |

Bold values indicate measured H_2_S below the suggested 0.165 mg L^-1^ protective limit for WR; *Defined as the measurable fraction of Fe which passed through a 0.45 µM pore-size filter and acidified to *p*H < 2.0 using trace metal grade HNO_3_; NM = not measured

**S.I. Table 4** Paddy A H_2_S, Fe, and SO_4_ in diffusion-based peeper PW samples (all mg L^-1^; n = 1 for each sampling event per location)

|  | Inflow | | | Middle | | | Outflow | | |
| --- | --- | --- | --- | --- | --- | --- | --- | --- | --- |
|  | Peeper | *Fe | SO_4_ | Peeper | *Fe | SO_4_ | Peeper | *Fe | SO_4_ |
| Jun. 04, 2019 | 0.280 | 76.7 | 985 | 0.449 | 12.1 | 41 | 2.68 | 3.13 | 476 |
| Jul. 02 | 0.344 | 25.9 | 659 | 0.338 | 11.2 | 3 | 0.726 | 5.12 | 245 |
| Aug. 01 | 0.703 | 14.8 | 234 | 3.04 | 9.59 | 107 | 0.747 | 1.52 | 204 |
| Sept. 03 | 2.41 | 2.33 | 417 | 1.72 | 7.57 | 163 | 0.595 | 2.54 | 78 |
| Oct. 03 | 0.670 | 1.92 | 315 | 0.477 | 5.98 | 175 | 1.54 | 1.07 | 177 |

*Defined as the measurable fraction of Fe which passed through a 0.45 µM pore-size filter and acidified to *p*H < 2.0 using trace metal grade HNO_3_

**S.I. Table 5** Paddy C H_2_S, Fe, and SO_4_ in diffusion-based peeper, and H_2_S in Rhizon (Rh), PW samples (all mg L^-1^; n = 1 for each sampling event per location)

|  | Inflow | | | | Middle | | | | Outflow | | | |
| --- | --- | --- | --- | --- | --- | --- | --- | --- | --- | --- | --- | --- |
|  | Peeper | Rh. | *Fe | SO_4_ | Peeper | Rh. | *Fe | SO_4_ | Peeper | Rh. | *Fe | SO_4_ |
| Jul. 02, 2018 | 0.221 | **0.118** | 15.8 | 595 | 0.576 | BD | 4.92 | 84 | 0.658 | **0.150** | 10.8 | 137 |
| Aug. 02 | 0.370 | **0.121** | 10.1 | 432 | 0.268 | **0.096** | 8.47 | 210 | 0.372 | **0.105** | 28.8 | 612 |
| Aug. 30 | 0.496 | 0.207 | NM | NM | 0.332 | BD | NM | NM | 0.377 | 0.314 | NM | NM |
| Oct. 01 | 0.529 | 0.182 | 4.99 | 383 | 0.398 | **0.134** | 6.22 | 205 | 0.340 | 0.271 | 23.5 | 378 |

Bold values indicate measured H_2_S below the suggested 0.165 mg L^-1^ protective level for WR; BD = below detection (< 0.078 mg L^-1^); *Defined as the measurable fraction of Fe which passed through a 0.45 µM pore-size filter and acidified to *p*H < 2.0 using trace metal grade HNO_3_; NM = not measured

**S.I. Table 6** Paddy C H_2_S, Fe, and SO_4_ in diffusion-based peeper PW samples (all mg L^-1^; n = 1 for each sampling event per location)

|  | Inflow | | | Middle | | | Outflow | | |
| --- | --- | --- | --- | --- | --- | --- | --- | --- | --- |
|  | Peeper | *Fe | SO_4_ | Peeper | *Fe | SO_4_ | Peeper | *Fe | SO_4_ |
| Jun. 04, 2019 | 0.31 | 7.55 | 441 | 1.7 | 12.4 | 574 | 0.7 | 11.9 | 129 |
| Jul. 02 | 0.423 | 4.79 | 199 | 0.525 | 5.73 | 187 | 0.739 | 5.68 | 90 |
| Aug. 01 | 0.848 | 0.642 | 468 | 0.706 | 0.974 | 350 | 6.03 | 0.368 | 358 |
| Sept. 03 | 0.682 | 0.38 | 322 | 0.571 | 1.63 | 320 | 0.443 | 4.61 | 326 |
| Oct. 03 | 0.802 | NS | 383 | 0.898 | 0.436 | 143 | 1.09 | 7.97 | 10 |

*Defined as the measurable fraction of Fe which passed through a 0.45 µM pore-size filter and acidified to *p*H < 2.0 using trace metal grade HNO_3_; NS = no sample
